# Relatively semi-conservative replication and a folded slippage model for short tandem repeats

**DOI:** 10.1186/s12864-020-06949-5

**Published:** 2020-08-17

**Authors:** Hongxi Zhang, Douyue Li, Xiangyan Zhao, Saichao Pan, Xiaolong Wu, Shan Peng, Hanrou Huang, Ruixue Shi, Zhongyang Tan

**Affiliations:** Bioinformatics Center, College of Biology, Hunan University, Changsha, 410082 China

## Abstract

**Background:**

The ubiquitous presence of short tandem repeats (STRs) in virtually all genomes implicates their functional relevance, while a widely-accepted definition of STR is yet to be established. Previous studies majorly focus on relatively longer STRs, while shorter repeats were generally excluded. Herein, we have adopted a more generous criteria to define shorter repeats, which has led to the definition of a much larger number of STRs that lack prior analysis. Using this definition, we analyzed the short repeats in 55 randomly selected segments in 55 randomly selected genomic sequences from a fairly wide range of species covering animals, plants, fungi, protozoa, bacteria, archaea and viruses.

**Results:**

Our analysis reveals a high percentage of short repeats in all 55 randomly selected segments, indicating that the universal presence of high-content short repeats could be a common characteristic of genomes across all biological kingdoms. Therefore, it is reasonable to assume a mechanism for continuous production of repeats that can make the replicating process relatively semi-conservative. We have proposed a folded replication slippage model that considers the geometric space of nucleotides and hydrogen bond stability to explain the mechanism more explicitly, with improving the existing straight-line slippage model. The folded slippage model can explain the expansion and contraction of mono- to hexa- nucleotide repeats with proper folding angles. Analysis of external forces in the folding template strands also suggests that expansion exists more commonly than contraction in the short tandem repeats.

**Conclusion:**

The folded replication slippage model provides a reasonable explanation for the continuous occurrences of simple sequence repeats in genomes. This model also contributes to the explanation of STR-to-genome evolution and is an alternative model that complements semi-conservative replication.

## Background

Short tandem repeats (STRs), also referred as simple sequence repeats (SSRs), have attracted increasingly great interests in recent decades [1–7], and have been widely analyzed in the sequences of eukaryotic, prokaryotic and also viral genomes [2, 5, 6, 8]. STRs are the most variable genomic sequences, which tend to appear frequent variations in repeat-unit number instead of nucleotide substitution, and they may be a critical power accelerate the genomic evolution [5, 9], have roles associate with the host-adaptation and pathogenicity [9, 10], be relevant with the expression of genes and activity of promoters [4, 11], have relationship with many genetic diseases [12–14], and be observed with microsatellite instability (MSI) in many type of cancers [15–18].

Though STRs have been comprehensively researched, there is actually no precise definition or wide-convinced standard for the extraction of STRs all the time, which is usually based on setting the minimum numbers of the iterations for the mononucleotide to hexanucleotide repeats based on empirical criterion [2, 3, 5, 9, 19, 20]. Majority of previous studies showed more interests into the relatively longer repetitive sequences [21–23], and most studies usually used the thresholds of 6, 3, 3, 3, 3, 3 for extracting mono- to hexanucleotide repeats [24–27], while the very short repeat-motifs with smaller iterations were almost excluded, causing the neglect of their important significance [28–31]. In this work, the selected STRs were extensively extracted with a wider extracting standard for extensive repeat-motif grabbing to investigate the essential occurrences of STR.

It is widely accepted that DNA slippage is thought to be the primary mechanism for driving STR expansion or contraction, however, slippage involves DNA polymerase pausing, dissociation and re-association [5, 32, 33], which may help to understand the expansion and contraction of long repeat sequences; it seems difficult to explain the remain of high percentage of short repeat sequences, and therefore, it is necessary to improve the slippage model more explicit to explain the generation of large amounts of short repeat sequences [34–37]. It was suggested that the STRs are most possibly born in the process of replication [5]; replication is considered to be exactly semi-conservative with that the number of nucleotides in replicating chain is be precisely equal to that in template chain, and the replicating DNA molecule was shown as a straight molecule in vitro [38, 39]. Though it is well known that the DNA molecule is highly bent and packed in a super helix state within the nucleus, the replicating DNA molecule was also believed to be dragged to a straight molecule by the polymerase complex in vivo [40–43]. But there are a lot of environmental elements inside the nucleus which may disturb the polymerase complex, and these disturbances sometimes may affect the dragged straight DNA molecule returning to some extent of bent. The bent replicating DNA molecule is possibly related to the polymerase slippage for the occurrence of short STRs. Here, we calculated the bent replicating DNA molecule with strictly considering the geometric space, the relationship between the phosphodiester bond and hydrogen bond, and also the stability of paired nucleotides; and proposed a folded replication slippage model for explaining repeats occurrence, which seems more reasonable to explain the remaining of high percentage short repeats in genomes, and also to explain the frequent STR expansion and contraction. This work may also put forward some constructive suggestions for complementing the theory of semi-conservative replication.

Here, we calculated the bent replicating DNA molecule with strictly considering the geometric space, the relationship between the phosphodiester bond and hydrogen bond, and also the stability of paired nucleotides; and proposed a folded replication slippage model for explaining repeats occurrence, which seems more reasonable to explain the remaining of high percentage short repeats in genomes, and also to explain the frequent STR expansion and contraction. This work may also put forward some constructive suggestions for complementing the theory of semi-conservative replication.

## Results

### Genomes tend to produce short repeats

We analyzed 55 randomly-selected sequence segments covering animal, plant, fungus, protist, bacteria, archaea and virus (Table S1). The STRs were extracted from all these sequence segments using a threshold with minimum length of 3 base pairs or nucleotides. Though 2 iteration of di-, tri-, tetra-, penta- and hexa- nucleotide repeat sequence are usually ignored in most previous studies [2, 5, 28, 29, 31], we found that the abundance of such repetitive sequences cannot be justified by the theory of random occurrence. Moreover, iteration of 3 to 5 of mononucleotide repeats also cannot be justified as random sequences. Therefore, we adapted a much more generous set of thresholds for the definition of short STRs as 3, 2, 2, 2, 2, 2 for mono-, di-, tri-, tetra-, penta-, hexa- nucleotide repeats, respectively. Aiming to analyze unexplored shorter simple repeats. The resulting sequences from this generous set of thresholds were compared with those from another two set of thresholds. As a control experiment to rule out unintentional amplification of noise, we generated mimic sequences with the same size and nucleotide composition to the corresponding 55 reported sequences.

**Fig. 1:**
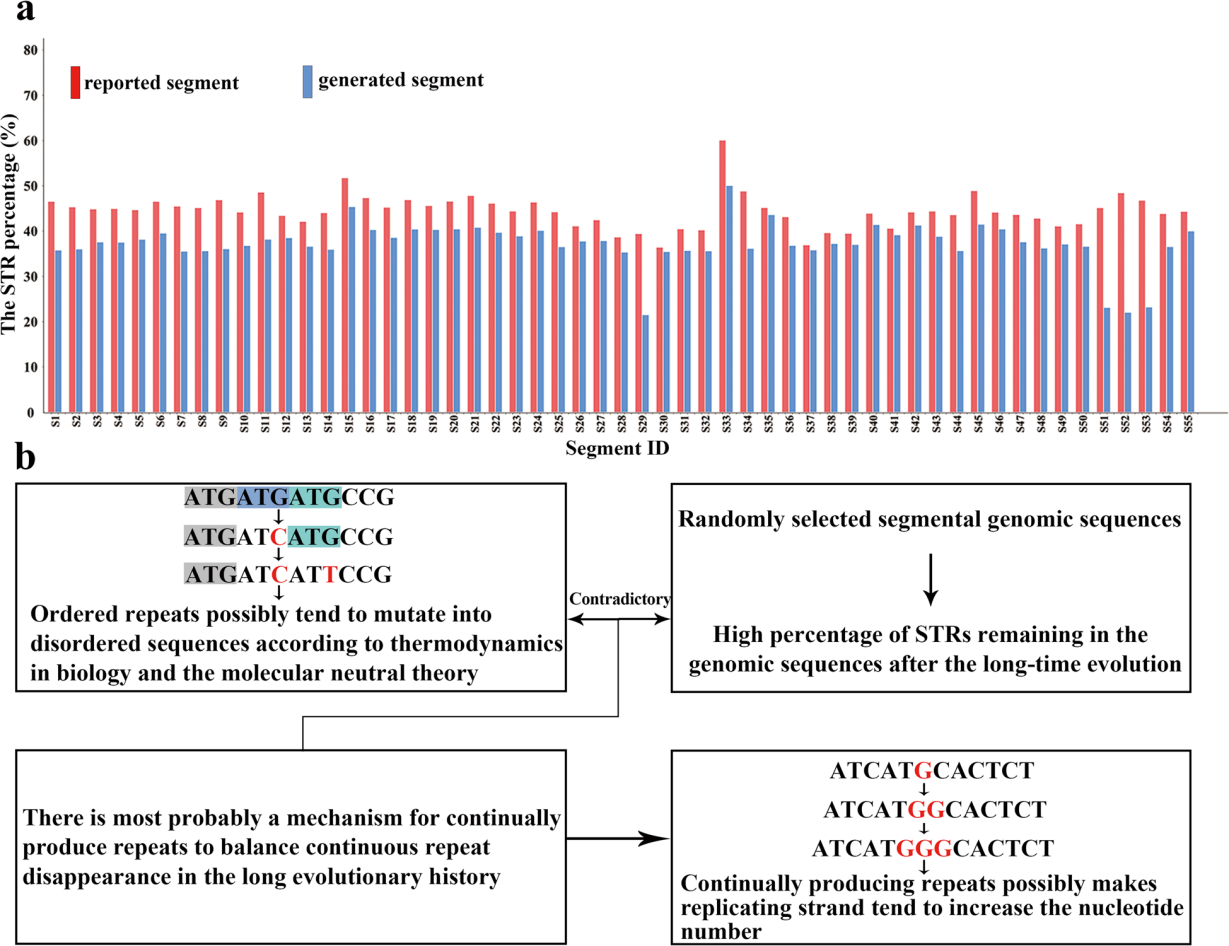
**A high** percentage of STRs in genomes and genomes probably tend to produce repeats. **a** STR percentages of 55 randomly-selected reported segments and the control group, which were the sequences generated with the same nucleotide numbers and components as those of the 55 selected reported segments but the random nucleotide orders by a program written in C language. **b** Contradiction analysis of disappearance and high percentage of STRs in the genomes

The analyzed data showed that the reported sequence segments comprise 36.4 to 60.0% STRs under the new threshold, with an average of 44.4% (Fig. 1a, Table S1), while comparative analysis using existing standards yielded only an average of 18.8 and 5.0% STR contents on the same dataset. Since all these segments were randomly selected from their genomes, our results suggested that the high content of short STRs is a general feature of all organism genomes after long time evolution, and that the few formerly well-studied repeats may only stand for the proverbial tip of the iceberg [2, 3, 5, 6, 8]. The null hypothesis test demonstrated that the percentages of STRs in the generated segments are all lower than those in the reported segments, indicating that the high percentages of short STRs preserved valuable signals in all reported segments.

Though the evolutionary mechanism of nucleotide sequences is still hotly debated by evolutionist, it is widely accepted that the mutation of genomic sequences occurs continually, persistently and permanently. The neutral molecular evolution and molecular clock theories suggest that the nucleotide substitution is constant over the course of evolution; while the thermodynamics in biology states that an isolated system tend to disorder [44–49]. According to the former stated theories, any ordered sequences such as STRs would mutate into disordered sequences in the long evolutionary history without the presence of selective pressure. This theory alone would result in the dilution of STRs and cannot explain the universal presence of preserved high content of STRs in genomes. Therefore, there is most probably an unexplored alternative mechanism for continually producing repeats to balance the continuous disappearance of STRs by random mutation, so as to maintain a high content of short repeat sequences in genomes across all biological kingdoms (Fig. 1b).

Furthermore, the STRs of small iteration numbers were observed to occur more frequently than those of large iteration numbers in all analyzed segments (Table 1, Table S2). A plausible explanation is that the STRs of small iteration numbers may be the basis for forming the STRs of large iteration numbers, otherwise, the STRs of large iteration numbers should occur as frequently as the STRs of small iteration numbers. Some of the longer STRs also possibly mutate into short STRs by contraction and point mutation as debated by many evolutionists [5, 13, 50], and these debates are possible because most short repeats were not considered in their statistics. On the contrary, our observations support the hypothesis that most of longer STRs evolved from the short STRs by expansion, and the genomes tend to produce short repeats by a continual mechanism with the preference of expansion against contraction.

**Table 1:** The lengths (bp) of STRs with different repeat unit types and different iterations in the segment of the reported human reference X chromosomal sequence at the location of 144,822–231,384 bp

Iteration	Mono^a^	Di	Tri	Tetra	Penta	Hexa	Total
I_2_	(18128)^b^	10,040	3540	2056	1250	480	17,366
I_3_	9702	1782	288	156	45	18	11,991
I_4_	3844	368	12	112	–	–	4336
I_5_	2095	120	15	20	–	–	2250
I_6_	600	24	18	0	–	–	642
I_7_	182	14	-^c^	28	–	–	224
I_8_	128	16	–	0	–	–	144
I_9_	54	18	–	36	–	–	108
I_10_	50	0	–	–	–	–	50
I_11_	55	22	–	–	–	–	77
I_12_	24	–	–	–	–	–	24
I_13_	65	–	–	–	–	–	65
I_14_	56	–	–	–	–	–	56
I_15_	45	–	–	–	–	–	45
I_16_	64	–	–	–	–	–	64
I_17_	0	–	–	–	–	–	0
I_18_	36	–	–	–	–	–	36
I_19_	19	–	–	–	–	–	19
I_20_	0	–	–	–	–	–	0
I_21_	42	–	–	–	–	–	42
I_22_	0	–	–	–	–	–	0
I_23_	23	–	–	–	–	–	23
I_24_	0	–	–	–	–	–	0
I_25_	25	–	–	–	–	–	25
I_26_	–	–	–	–	–	–	–
I_27_	–	–	–	–	–	–	–
I_28_	–	–	–	–	–	–	–
Sum	17,109	12,404	3873	2408	1295	498	37,587

^a^ Mononucleotide repeat (Mono), Dinucleotide repeat (Di), Trinucleotide repeat (Tri), Tetranucleotide repeat (Tetra), Pentanucleotide repeat (Penta), Hexanucleotide repeat (Hexa)

^b^ The length of mononucleotide repeats with iterations of 2 was not included in this statistics and just used as the reference here

^c^ Beyond the largest iteration of this repeat unit type in corresponding analyzed segments were expressed as “-“

### Relatively semi-conservative replication

It is well known that each base pair of DNA is a one-to-one correspondence without other extra residue during replication in the double-helix model [38, 39]. And Meselson and Stahl have verified that the replication of DNA chains is semi-conservative by sedimentation techniques based on the diversity differential of DNA with different isotopes, implicating that the number of nucleotides in the replicating strand is consistent with that in the template strand during a complete replication process [51]. However, if the preserved high content of short repeats is produced during replication as described above, the number of nucleotides in the replication strand would be one or several nucleotides/motifs higher than that in the template strand. In vitro experiments also revealed the presence of repeats during DNA replication, and the nascent replication chain has an increase in the number of nucleobases [30, 40, 41, 52]. In the case of our relatively semi-conservative replication model, the replication process can be described as the following formula:

1 $$ {N}_i=\operatorname{int}\left[{N}_0\left(1+{f}_1{\lambda}_1\right)\left(1+{f}_2{\lambda}_2\right)\dots \left(1+{f}_i{\lambda}_i\right)\right] $$

2 $$ \varDelta {N}_i={N}_i-{N}_{i-1}=\operatorname{int}\left[{N}_0{f}_i{\lambda}_i\left(1+{f}_1{\lambda}_1\right)\left(1+{f}_2{\lambda}_2\right)\dots \left(1+{f}_{i-1}{\lambda}_{i-1}\right)\right]\ge 0 $$

*N*_*0*_: The number of nucleotides in the initial template strand;

*N*_*i*_: The number of nucleotides in the replicating strand during No. *i* round replication;

int[]: Round the value to the lower integer;

*ΔN*_*i*_: The difference of the nucleotide numbers between *N*_*i*_ and *N*_*i-1*_;

*λ*_*i*_ (*λ*_*i*_ → 0): The coefficient of occurring repeats during No. *i* round replication; and is most probably an infinitesimal relating to the possibility of repeat occurrence;

*f*_*i*_ (0 ≤ *f*_*i*_ ≤ 1): The fixation coefficient of repeat sequences during No. *i* round replication.

In general, the number of nucleotides in the replicating strand is likely to have exactly equal to that in the template strand. This observation is consistent with our model when the observed template strand is short and the number of replication rounds is relatively low. For example, the total number of nucleotides in the initial template strand for stable PCR is up to two to three thousand nucleotides. When we suppose *N*_0_ = 3000, *λ*_*1*_ = 10^− 5^, *f*_*1*_ *=* 1, the value of Δ*N*_*1*_ would be 0 according to the formula (2), and therefore, *N*_*1*_ = *N*_*0*_, causing the replicating strand to be no longer (or no shorter) than the template strand, and the discovery of nascent repeat is unavailable. Nevertheless, when the observed strand is long enough to result in a *ΔN*_*i*_ of larger than 1, our model would explain how the number of nucleotides in the replicating strand changes from that in the template strand. For instance, when we suppose *N*_0_ = 10^6^, *λ*_*1*_ = 10^− 5^, *f*_*1*_ *=* 1, the value of Δ*N*_*1*_ would be 10, which could result in the increase of 10 nucleotides (or repeat-motifs) in the replicating strands when compared with the template strand. The increased number of nucleotides may represent nascent repeat sequences according to our relatively semi-conservative replication model.

The occurrence of STRs would possibly encounter selective pressure, though it may be different in coding or non-coding regions. We use *f*_*i*_ to represent the fixation possibility of the nascent repeats under selective pressure. A fixation coefficient of 0 (*f*_*i*_ = 0) indicates the occurrence of nascent repeats that are lethal mutations and unable to produce survivable offspring, or may be excluded by the DNA repair system [1, 53]. A fixation coefficient of 0 < *f*_*i*_ < 1 indicates that the nascent STRs are deleterious but still can be fixed in the genome with survived offspring, like Huntington’s disease [14]. A fixation coefficient of 0 ≤ *f*_*i*_ ≤ 1 also includes the cases with occurrences of nascent STRs being neutral mutations, which can be either retained or excluded depending on genetic drift. A fixation coefficient of 1 (*f*_*i*_ = 1) indicates beneficial mutations, representing that the nascent STRs may help the organism surviving. Therefore, the preserved high content of short repeats suggests that the replicating process frequently produce short repeat sequences which may be fixed neutrally, beneficially, or deleteriously with diseases. This suggests that the replication process may be relatively semi-conservative.

### Folded slippage model

**Fig. 2:**
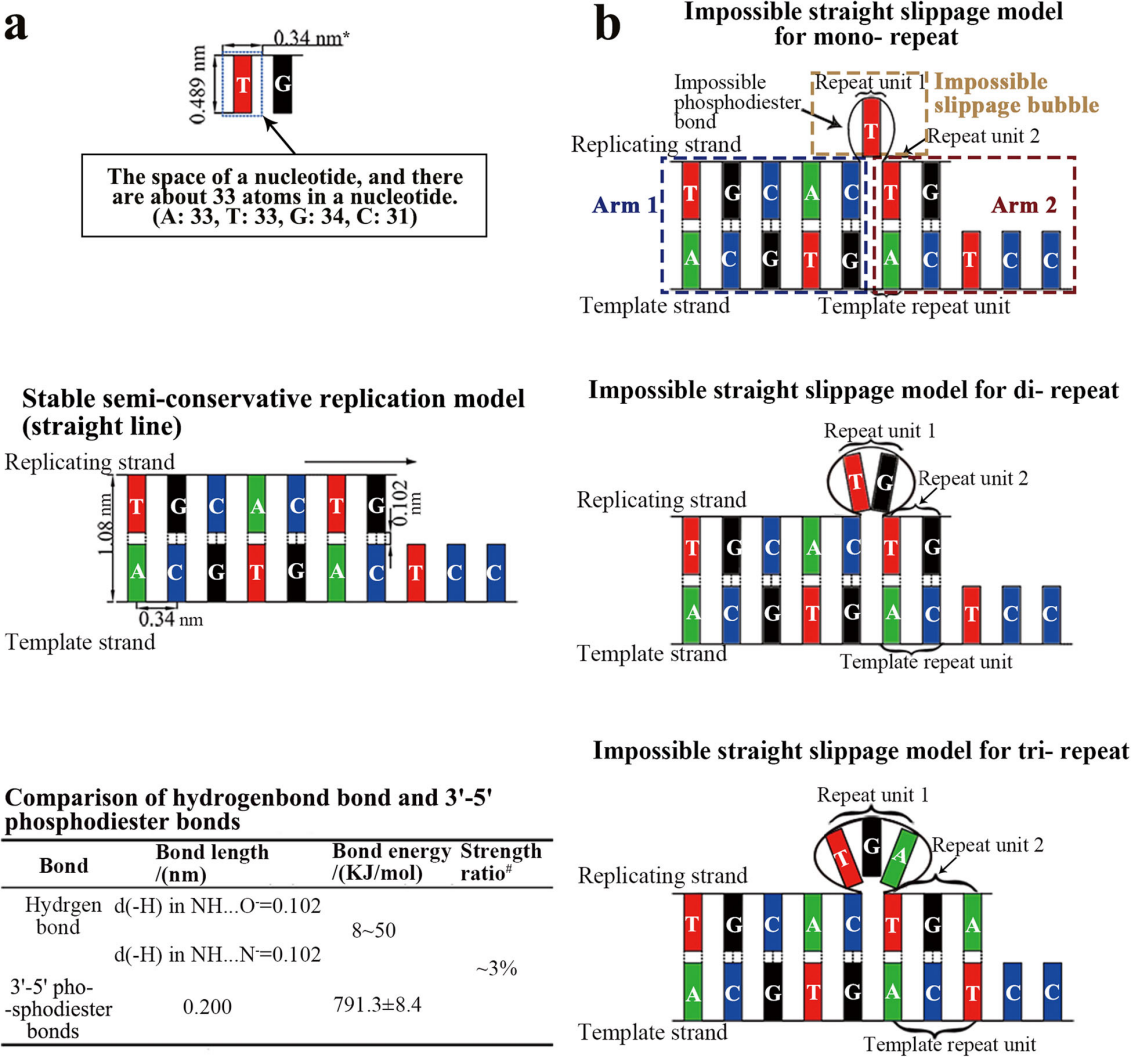
Straight strand models of semi-conservative replication and slippage. **a** The space of a nucleotide was drawn. * indicates that those number is the theoretical values (top); The stable straight model of semi-conservative replication (middle); The comparison of hydrogen bond and 3′-5′ phosphodiester bonds (bottom) [55–57]. ^#^ indicates the strength ratio was calculated by the strength of hydrogen bond dividing that of phosphodiester bond. **b** The impossible straight slippage models of mononucleotide, dinucleotide and trinucleotide repeats according to the strict geometric calculation of the space of a nucleotide and the stability of hydrogen and phosphodiester bonds

The nucleotide chains of various species tend to produce simple repeats, which is likely to be caused by the insertion of additional nucleotides during the replication process. However, the mechanism by which simple repeats actually form during the replication process is still highly debated [5, 50, 54]. The widely accepted mechanism of occurring STR is the replication slippage model, which could explain the expansion and contraction of longer STRs, but not the expansion and contraction of much amounts of short repeats. The existing slippage model is indeed a straight template strand model, with no plausible consideration regarding the space required for the nascent nucleobase, the much stronger phosphodiester bonds when compared with hydrogen bonds (Fig. 2a) [55, 56], and the force that drives the replicate strand slippage. The straight replication slippage model suggests that the STRs possibly occurred by slippage occasionally [13, 58–60], but is rather ambiguous about further details in the mechanism. Actually, there are about 33 atoms in a nucleotide (A: 33, T: 33, G: 34, C: 31) [61], which possess a certain physical space in the molecule. According to previous reports, we simplified a nucleotide space into an intuitive plane model, whose length is about 0.489 nm (length = (distance between the double helix 1.08 - Hydrogen bond length 0.102) / 2), and with a width of 0.34 nm which is the distance between each pair of bases (Fig. 2a) [55–57]. We reconstructed the linear replication slippage model with a CAD geometric calculation by considering the space of bases (Fig. 2b, Fig. S1). If the slippage bubble has enough geometric space to accommodate the repeat unit, the phosphodiester bond would be stretched to far more than 0.34 nm. This is contradictory to the chemical principle that the phosphodiester bonds in DNA is actually much stronger than the hydrogen bonds (Fig. 2a) [57]. Since it is impossible to form a slippage bubble by a larger elongation of the phosphodiester bonds to accommodate the nascent repeat unit, the straight slippage model is insufficient to explain to the occurrence of short repeats and a more sophisticated slippage model should be proposed.

**Fig. 3:**
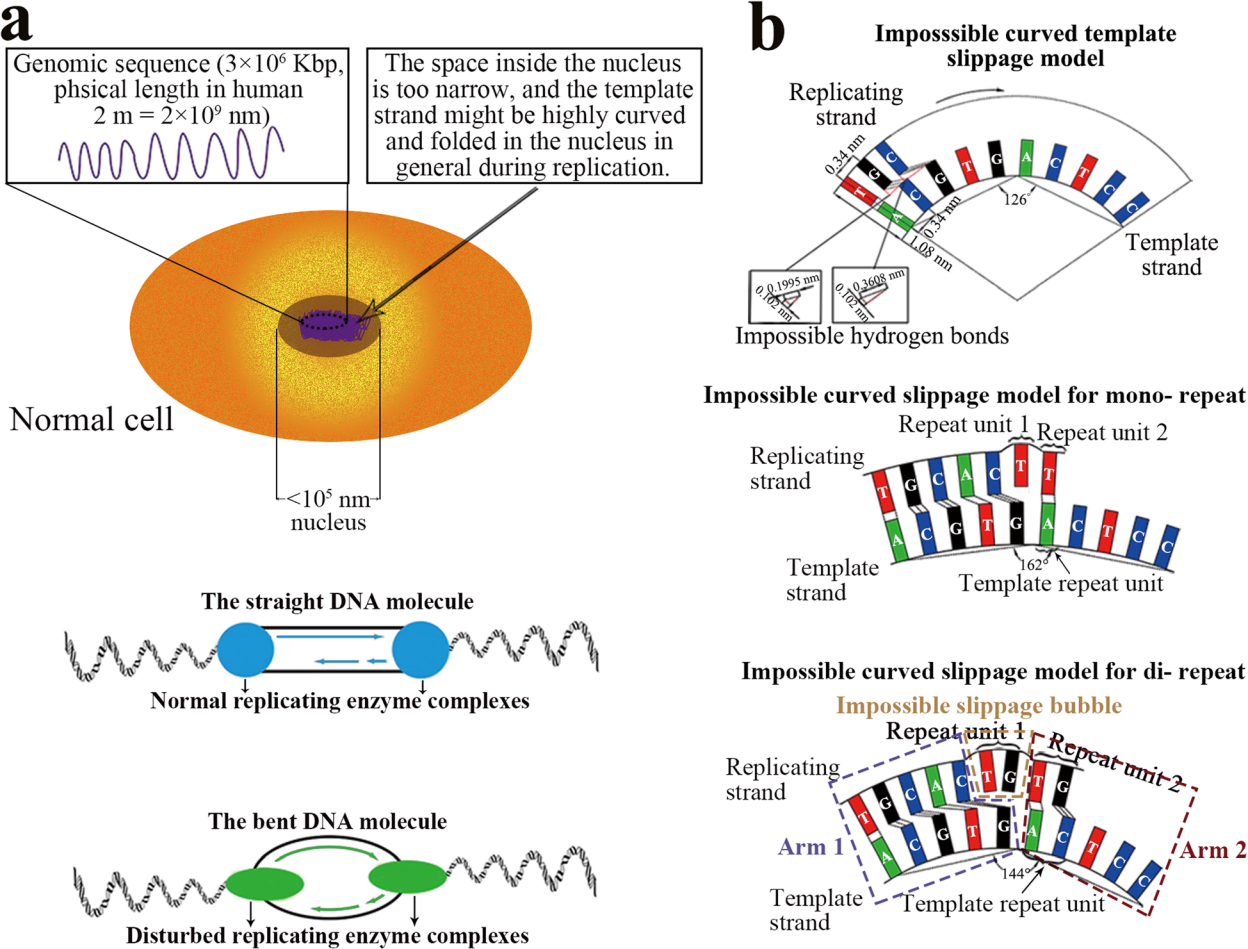
The DNA chain is highly curved or folded in the nucleus and the impossible curved slippage model. **a** Schematic diagram of the size of the nuclear space (top) [61]; The normal replicating enzymes complex straighten the DNA chain, while the disturbed replicating enzymes complex may cause the DNA molecule return to curved state (bottom). **b** Impossible curved template slippage model according to the strict geometric calculation of the space of a nucleotide and the stability of hydrogen and phosphodiester bonds (top); Mono- and dinucleotide repeats may be impossibly produced in curved replicating strands (middle and bottom)

Actually existing replication slippage studies has largely overlooked the validity of the straight template strand assumption in the replication process – the template strands are thought to be perfectly straight in all replication models. Though the template strands are indeed straight in general condition, the possibility of a kinked strand cannot be ruled out. It is well known that the dimension of fully unfolded and extended genomic DNA chains are several magnitudes higher than the dimension of the nucleus (Fig. 3a). For example, the total length of human genome is about 2 m (2 × 10^9^ nm), while the diameter of nucleus is beneath 10^5^ nm in human cell [61]. Therefore, the genomic DNA chains are generally highly compacted and folded in the nucleus. During the semi-conservative replication, the replicating molecule is believed to be a straight molecule [40–43], while the replicating enzyme complexes usually straighten the template strand and make the replicating strand well paired with the template strand [40, 62, 63]. However, environmental factors such as temperature, viral proteins or diseases may disrupt the normal works of the enzyme complexes. We speculate that such disruption of the enzyme complex may cause both the replicating strand and the template strand to regain their curved or folded state, resulting in the emergence of provisional kinked strands.

First, we proposed a curved template slippage model for the replication process. When the curved DNA strand is used as the template strand on the inner side, the replication strand is longer than the template strand and can form more nucleotides than the template strand on the outer side. The replication strand should be longer than the template strand so as to provide extra spaces for accommodating the extra repeat bases (Fig. 3b). The links of base pairs mainly depend on 2 types of hydrogen bonds, N—H …: N and N—H …: O [55], with a strength at about 3% of the 3′, 5′-phosphodiester bonds [56, 57, 64, 65] (Fig. 2a). While the distance between the bases is fixed at the backbone, the strengths of the hydrogen bonds are negatively correlated to the distance between every base pair. Therefore, the curved template slippage model would cause the hydrogen bonds to exceed the threshold of 0.167 nm and break off [55]. The curved slippage model partially explains the spaces that form slippage bubble, yet at the cost of forming unstable hydrogen bonds double-chain structures (Arm1 and Arm2) on both sides of the slippage bubble (Fig. 3b, Fig. S2). The curved slippage model is an advance from the classic straight slippage model but still has fundamental flaw.

**Fig. 4:**
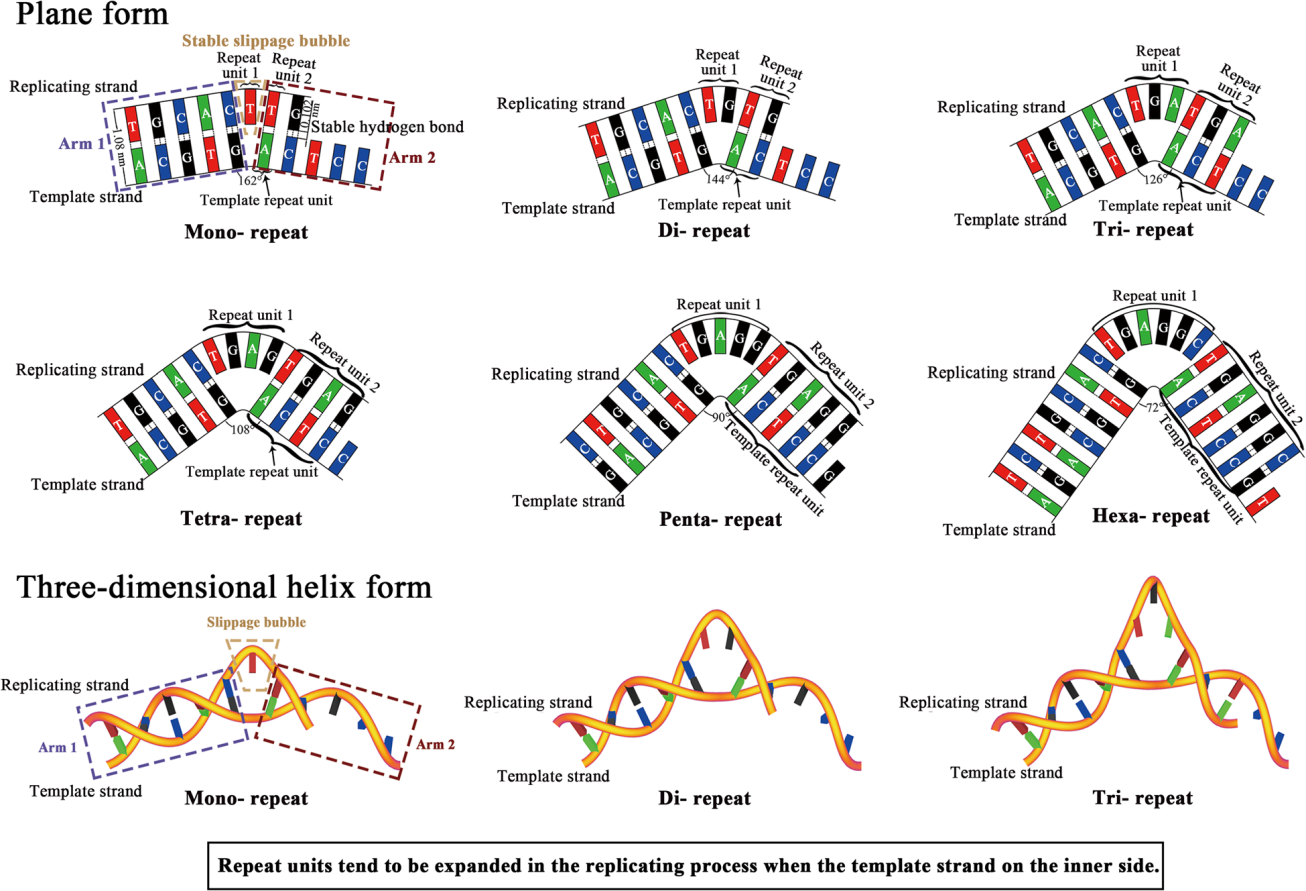
Stable folded slippage models of mononucleotide to hexanucleotide repeats amplification according to the strict geometric calculation of the space of a nucleotide and the stability of hydrogen and phosphodiester bonds. Repeat units tend to be expanded in the replicating strands when the template strands are on the inner side of the folded slippage models respectively. The bottom 3 sub-figures were the folded slippage models in three-dimensional helix form

**Fig. 5:**
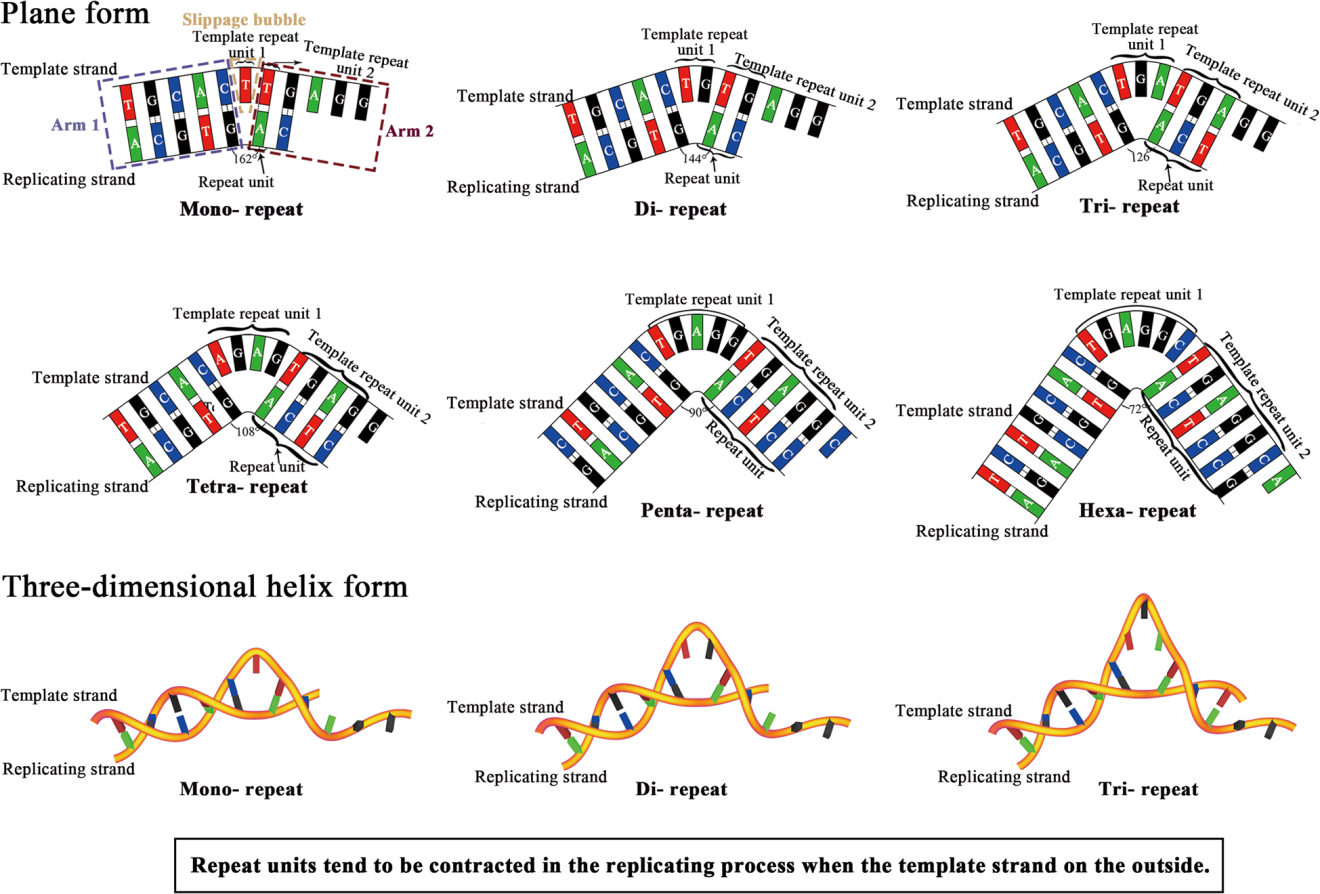
Stable folded slippage models of mononucleotide to hexanucleotide repeats contraction according to the strict geometric calculation of the space of a nucleotide and the stability of hydrogen and phosphodiester bonds. Repeat units tend to be subtracted in the replicating strands when the template strands are on the outside of the folded slippage models respectively. The bottom 3 sub-figures were the folded slippage models in three-dimensional helix form

Then we proposed a folded slippage model. The folded template strand forms a slippage bubble above the folding site to accommodate the repeat nucleotides during the replication process. The phosphodiester bonds are fixed and the bases are well paired with stable hydrogen bonds on both sides of the slippage bubble (Fig. 4). With proper folding angle, a stable double-stranded folded slippage structure can provide chances to produce repeats, while satisfying factors including sufficient nucleotide geometric spaces, stable phosphodiester bonds and stable hydrogen bonds. Actually, there are two variations of the folded slippage models: When template strand is on the inner side, the repeat unit duplicates to produce new repetitive unit or repeat expansion (Fig. 4); and when the template strand is on the outer side, the replication strand may make the repetitive sequences to contract (Fig. 5). The features of this folded slippage model can explain the widely observed STR mutations with expansion and contraction of repeat units [5, 13, 50, 59, 66]. In addition, replication slippage of template strands with different folding angles may result in the expansion or contraction of repeat units with different sizes. When template chains are folded on the inner side at a folding angle of 18°, 36°, 54°, 72°, 90° and 108°, the replication strands would produce mono-, di-, tri-, tetra-, penta-, hexa-nucleotide repeat expansions, respectively (Fig. 4). With fixed phosphodiester bond, it is necessary to break off more hydrogen bonds to produce higher number of repeats. For example, since 2 or 3 hydrogen bonds are used to stabilize each base pair, 12–18 hydrogen bonds need to be broken to produce hexanucleotide repeats. This suggested that the difficulty to form repeats from mono- to hexanucleotide gradually increases, which explains our statistic data in which the occurrence of mono-, di-, tri-, tetra-, penta- and hexanucleotide repeat gradually decreases (Table 1, Table S2). Similarly, when template chains are folded on the outer side at a rotation angel of 18°, 36°, 54°, 72°, 90° and 108°, the replication strands will produce corresponding repeat contractions respectively (Fig. 5). These features of our folded slippage model can explain the emergence of short tandem repeats which usually refers to the tandem repeats with repeat units from mono- to hexanucleotides [5, 22, 27]. According to this rule, we also describe the possible folded template slippage models of hepta-, octa-, nona- and deca- nucleotide repeats (Figs. S3 and S4), while the replicating strand must break off 14–21, 16–24, 18–27, 20–30 hydrogen bonds to make a folded slippage bubble, respectively. Such long tandem repetitive sequences are unlikely to occur since the energy to break off 14–30 hydrogen bonds are on the same scale as the energy to break off one phosphodiester bond, which explains the observations that they are often much less abundant in the genomes [59, 67]. Our folded slippage model can also explain how the (A_m_T_n_) repeats tend to grow faster than (G_m_C_n_) repeats because smaller number of broken hydrogen bonds in the (A_m_T_n_) repeats impose lower energy barrier for repeat expansion [21, 37, 68, 69]. Although this folded slippage model is a simplified model described in a plane form, it simulates and explains the repeat sequences producing process. We also build a simplified double-helical model in three-dimensional forms to show the folded slippage model more intuitively (Figs. 4 and 5), while the precise folding angle and other issues deserve further study.

When compared with the straight template slippage model, the folded template model exhibits enough geometric space in the slippage bubble to accommodate repeat nucleotides without stretching the phosphodiester bonds. When compared with the curved template model, the folded model has two sides of the slippage bubble stably paired, and has Arm1 and Arm2 similar to the straight template replication model at both sides (Figs. 4 and 5). The folded model takes full account of the space required by nucleotides, the stability of phosphodiester bonds, and the strength comparison between phosphodiester bonds and hydrogen bond. This model can explain STR mutations with repeat unit expansion and contraction, and provides a plausible explanation for the production of short repeats production in the replicating process which otherwise neither the straight slippage model nor the curved slippage model can explain. The folded template strand slippage model may be responsible for the continual production of repeat sequences and the retention of high percentage of repeat sequences in genomes.

## Discussion

According to the folded slippage model, the template chain folding on the inner side may make the replicating chain slippage for repeat expansion, while the template chain folding on the outer side may make the replicating chain slippage for repeat contraction. At a first glance, the possibility of repeat expansion and contraction may appear to be the same. However, there are two manners for the repeat sequences contraction, one is above mentioned the template chain folds on outside, another is also general mutations stated above. The high content of repeat sequences is still in a stable state in the genome of each species, implicating a higher rate for repeat expansion when compared with repeat contraction, which is also reported in many other studies [30, 52, 70].

**Fig. 6:**
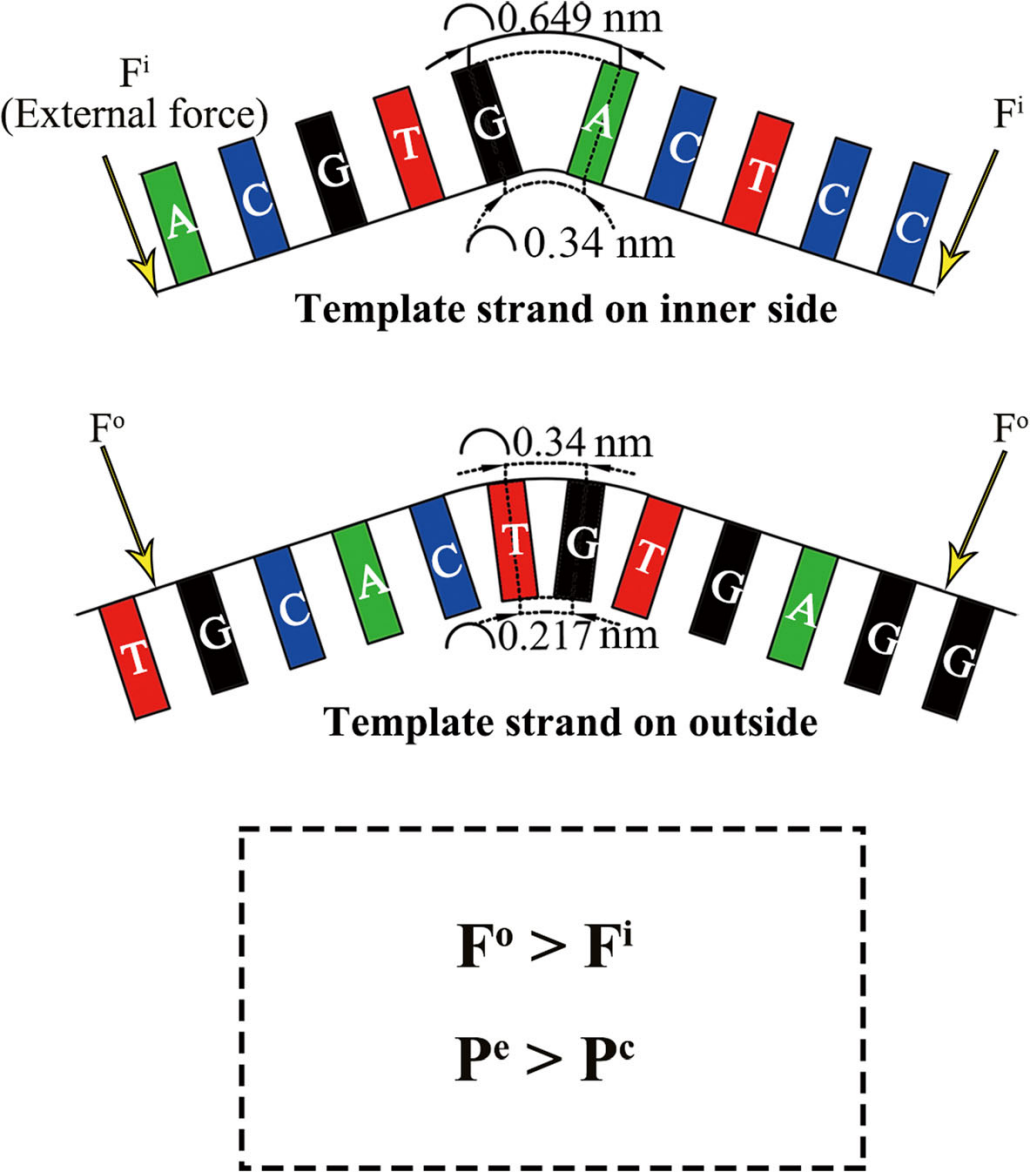
Repeat production incline to expansion. F^o^, F^i^ refer to the force required for the two template strands to bend, respectively. F^o^ > F^i^ means that the force of the template strand bending downward is greater than the bending upward, and P^e^ > P^c^ means that the possibility of the template strand bending upward is greater than the downward bending

Under normal circumstances, the replicating enzyme complexes may provide power for balancing the external forces to drag the template DNA molecule straight. However, when the replicating enzyme complexes are disturbed, the replicating straight template DNA chain should return to folded under external forces from the narrow and crowded cell nucleus. We proposed an external force model for template strand returning to folded, and this model may be helpful to explore the probability of expansion and contraction. When the template strand is on the inner side, the nucleotide bases point outward, and the space of bases at the folded site become wide and loose at outward part; while it is on the outer side, the base in the folding position is squeezed inward. Comprehensive consideration of the small difference of the space of nucleotides at the folded site reveals that the external forces to make template strand folded with bases loose should be smaller than that with base squeezed. Therefore, the external force required for the template strand folded on the outside (F^o^) is inevitable greater than that on the inner side (F^i^). F^o^ > F^i^ suggests that the probability for the template strand folded on the inner side is higher than that on the outer side. Our folded slippage model suggested that the repeats tend to expand when the template strand is on inner side and tend to contract when the template strand is on the outer side. Therefore, the odds of repeat expansion (P^e^) is higher than that for repeat contraction (P^c^), which can be described as P^e^ > P^c^ (Fig. 6). The STR studies, like in Huntington disease related locus and myotonic dystrophy type 1 locus, all showed STR expansion biased [13, 14, 71–73], which proves that the expansion of short STRs are more frequent than that of contraction.

Thus, according to formula (2):

When the template strand on the outer side, repeats tend to contract, so *λ*^*c*^ < 0,

thus, *ΔN*^*c*^ = *N*^*c*^_*i*_ − *N*^*c*^_*i* − 1_ = int[*N*_0_*f*^*c*^_*i*_*λ*^*c*^_*i*_(1 + *f*^*c*^_1_*λ*^*c*^_1_)(1 + *f*^*c*^_2_*λ*^*c*^_2_)…(1 + *f*^*c*^_*i* − 1_*λ*^*c*^_*i* − 1_)] ≤ 0.

When the template strand on the inner side, repeats tend to expand, so *λ*^*e*^ > 0,

thus, *ΔN*^*e*^ = *N*^*e*^_*j*_ − *N*^*e*^_*j* − 1_ = int[*N*_0_*f*^*e*^_*j*_*λ*^*e*^_*j*_(1 + *f*^*e*^_1_*λ*^*e*^_1_)(1 + *f*^*e*^_2_*λ*^*e*^_2_)…(1 + *f*^*e*^_*j* − 1_*λ*^*e*^_*j* − 1_)] ≥ 0.

The general repeat expansion and contraction can be described as:

$$ \kern12em {\displaystyle \begin{array}{c}\mid \sum \varDelta {N}^e\mid =\mid \operatorname{int}\left[\sum {N}_0{f^e}_j{\lambda^e}_j\left(1+{f^e}_1{\lambda^e}_1\right)\left(1+{f^e}_2{\lambda^e}_2\right)\dots \left(1+{f^e}_{j-1}{\lambda^e}_{j-1}\right)\right]\mid; \\ {}\mid \sum \varDelta {N}^c\mid =\mid \operatorname{int}\left[\sum {N}_0{f^c}_i{\lambda^c}_i\left(1+{f^c}_1{\lambda^c}_1\right)\left(1+{f^c}_2{\lambda^c}_2\right)\dots \left(1+{f^c}_{i-1}{\lambda^c}_{i-1}\right)\right]\mid; \\ {}\\ {}\sum \varDelta N=\mid \sum \varDelta {N}^e\mid -\mid \sum \varDelta {N}^c\mid =\operatorname{int}\left[\sum \left[\begin{array}{c}\mid {f^e}_j{\lambda^e}_j\left(1+{f^e}_1{\lambda^e}_1\right)\left(1+{f^e}_2{\lambda^e}_2\right)\dots \\ {}\dots \left(1+{f^e}_{j-1}{\lambda^e}_{j-1}\right)\mid \end{array}-|{f^c}_i{\lambda^c}_i\left(1+{f^c}_1{\lambda^c}_1\right)\left(1+{f^c}_2{\lambda^c}_2\right)\dots \left(1+f{\lambda^c}_{i-1}{\lambda^c}_{i-1}\right)|\right]{N}_0\right].\end{array}} $$

Because *λ* was defined as coefficient of occurring repeats, the possibility of repeat expansion (P^e^) is positively proportional to *λ*^*e*^ and the possibility of contraction (P^c^) is positively proportional to the absolute value of *λ*^*c*^ (|*λ*^*c*^|). Under the assumptions that *f*^*e*^ *= f*^*c*^ = *f*, *i* = *j*, and as generally P^e^ > P^c^, then *λ*^*e*^ > |*λ*^*c*^|, and also ∑[|*λ*^*e*^_*j*_(1 + *fλ*^*e*^_1_)(1 + *fλ*^*e*^_2_)…(1 + *fλ*^*e*^_*j* − 1_)|] ≥  ∑ [|*λ*^*c*^_*i*_(1 + *fλ*^*c*^_1_)(1 + *fλ*^*c*^_2_)…(1 + *fλ*^*c*^_*i* − 1_)|],

therefore, ∑*ΔN* = |∑*ΔN*^*e*^| − |∑*ΔN*^*c*^| ≥ 0.

So, when the external forces for returning the folded template strand were considered, the possibility of repeat expansion should be higher than that of repeat contraction, then the revised formula (2) is also able to explain the retention of high percentage of short repeats in genomes under a mechanism of continually producing repeats. This mechanism might result from the folded template chain slippage model, which is possibly responsible for the widely occurring STRs in eukaryotic, prokaryotic and also viral genomes. We improved the straight slippage model to a folded slippage model by fully considering the geometric spaces of nucleotide bases, the relationship between phosphodiester and hydrogen bond, and the stability of these bonds. The slippage model showed that the straight replicating template DNA may partially regain its folded state resulting from disturbed replicating enzyme complexes, and may provide chances for continually producing much amount of short repeats; though the long unit repeats may be explained by the former slippage model [33, 59].

The easily forming of folded slippage may also be responsible for the widely observed fact that repetitive part of genome is usually evolved one hundred or more times than other parts with only repeat unit expansion and contraction [1, 18, 50, 74], though the repeats occurred more in non-coding regions than in coding regions possibly due to different selective pressures [5, 13, 59]. Most of the emerging repeats should be lethal mutation and may have been negatively selected to lost; some of emerging repeats should be deleterious in genomes and responsible for a series of diseases [72, 73, 75, 76]; many neutral repeat expansions may be lost or fixed with no functions in genomes by genetic drift [77]; and some beneficial repeat expansions may promote the emergence of different new properties or functions – all of which lead to the abundance of repeat sequences in the genomes with a diversified set of roles as reported in the literature [9–11, 66, 68, 78, 79]. The longer repeats might originate from continuous short repeat expansion by the folded template slippage; the longer genomes possibly evolved from the short genomes in the long evolutionary replicating process.

## Conclusions

The universal presence of high-content short repeats is possibly a common characteristic of genomes across all biological kingdoms, which indicates a mechanism for continuous production of repeats. We proposed a folded replication slippage model, which provides a reasonable explanation for the continuous occurrences of STRs and their high contents in genomes with improving the existing straight-line slippage model, and this folded replication slippage model also suggests that expansion exists more commonly than contraction in the STRs without the presence of selective pressure. This model also contributes to the explanation of STR-to-genome evolution and is an alternative model that complements semi-conservative replication.

## Methods

### Sequences resource

We randomly selected 50 species covering animals, plants, fungus, protozoa, bacteria, archaea and viruses, according to the list of “KEGG Organism: Complete Genome” [80]. To simplify the analyses and make the analyzed data statistically representative, we randomly chose 55 sequence segments with size range from 3000 to 96,600 bp; the segments are out of 55 full genomic sequences from the 50 selected species, in which 5 species were randomly selected with double genomic sequences and 45 species were randomly selected with single genomic sequence from the reported data in Genbank; the segments were selected randomly in position and avoided to select incompletely sequenced gaps; the accession numbers with the related information were listed in Table S1.

### Repeat extraction

The perfect simple sequence repeats were extracted by Imperfect Microsatellite Extraction Webserver [81] from those 55 randomly selected segments. The minimum iterations for all perfect mono- to hexanucleotide repeats were set at 3, 2, 2, 2, 2, 2 to mine the data more completely in this study, comparing with most researchers setting iterations at relatively higher self-defined values, and 3 iterations for mononucleotide repeats were defined to ensure to be commonly recognized as the STRs.

### Null hypothesis test

We also extracted perfect mono- to hexanucleotide repeats under the above threshold in the sequences that were generated by a program written in C language (Program S1). The nucleotide compositions and numbers of the generate segments were the same as those of the selected segments, however, the nucleotide orders of the generate segments were randomly rearranged in the C program. Then, the validating test, which can verify that the short STRs extracted in those 55 reported segments are not randomly occurred, was based on the comparison of the STR percentages in the reported segments and the generated segments.

### Model drawing of DNA replication

Different models were drawn to simulate the DNA replication. Normally in straight model, the hydrogen bond length between 2 paired nucleotides is reported to be 0.102 nm and the distance between 2 neighboring nucleotides is 0.34 nm, importantly, owing to the nucleotides occupying almost same space in DNA strands, the space of a nucleotide was simplified into a geometric plane form in this analysis, which was 0.489 nm in length and 0.34 nm in width. Then we applied AutoCAD [82] to draw the straight, curved and folded slippage models according to the strict geometric calculation of the spaces of nucleotides and different strengths between hydrogen bonds and phosphodiester bonds. And the slippage models in helix structure were achieved by Rhino [83], which is an industrial drawing software.

## Supplementary information

**Additional file 1: Table S1.** The basic segment information and percentage comparison of short repeats between the randomly selected segments and the comparison of short repeats between the randomly selected segments and the corresponding generated segments.

**Additional file 2: Table S2.** The total lengths (bp) of STR with different repeat units and different iterations in 55 analyzed segmental sequences under the standard of 3, 2, 2, 2, 2, 2

**Additional file 3.** Supplementary Program

**Additional file 4.**

**Additional file 5.**

**Additional file 6.**

**Additional file 7.**

## Data Availability

The information including accession number of 55 analyzed sequence segments is listed in Table S1 and also available in the following git-hub web link. Table S2 is the dataset of STRs with different repeat units and different iterations in 55 analyzed segmental sequences under the standard of 3, 2, 2, 2, 2, 2, which are also available in the following git-hub web link. Program S1 for generating segments, Table S1 and Table S2 can be downloaded from https://github.com/DooYal/Supplementary-materials-for-submitting-relatively-...-.

## Abbreviations

SSR: Simple sequence repeat
SSRs: Simple sequence repeats
STR: Short tandem repeat
STRs: Short tandem repeats
