# Supplementary figures and images for: Relatively semi-conservative replication and a folded slippage model for short tandem repeats
Source: BMC Genomics. 2020 Aug 17;21:563. doi: 10.1186/s12864-020-06949-5 (PMC7430839; doi:10.1186/s12864-020-06949-5)

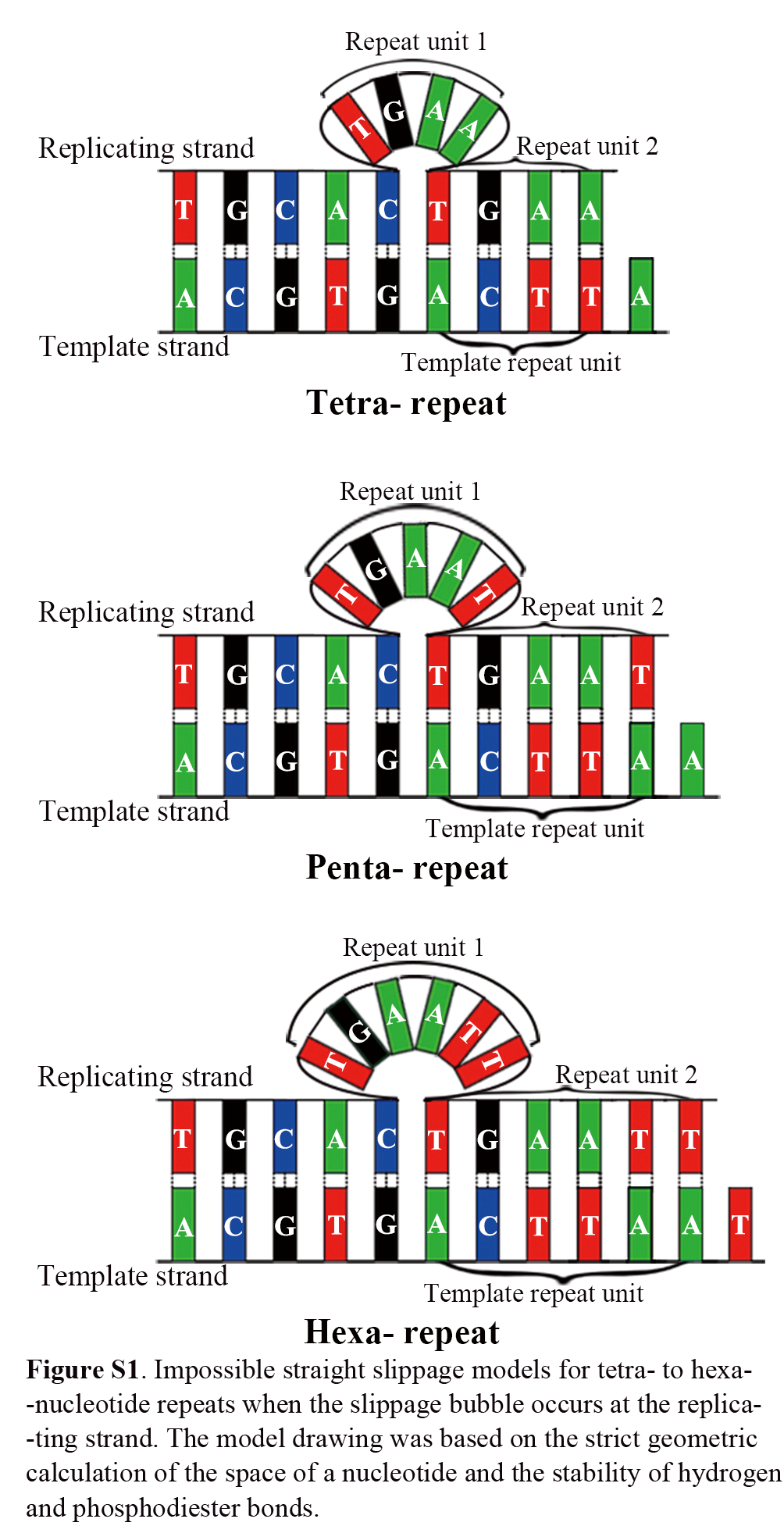

Supplement: Supplementary file 4 — Additional file 4. [file 12864_2020_6949_MOESM4_ESM.tiff]

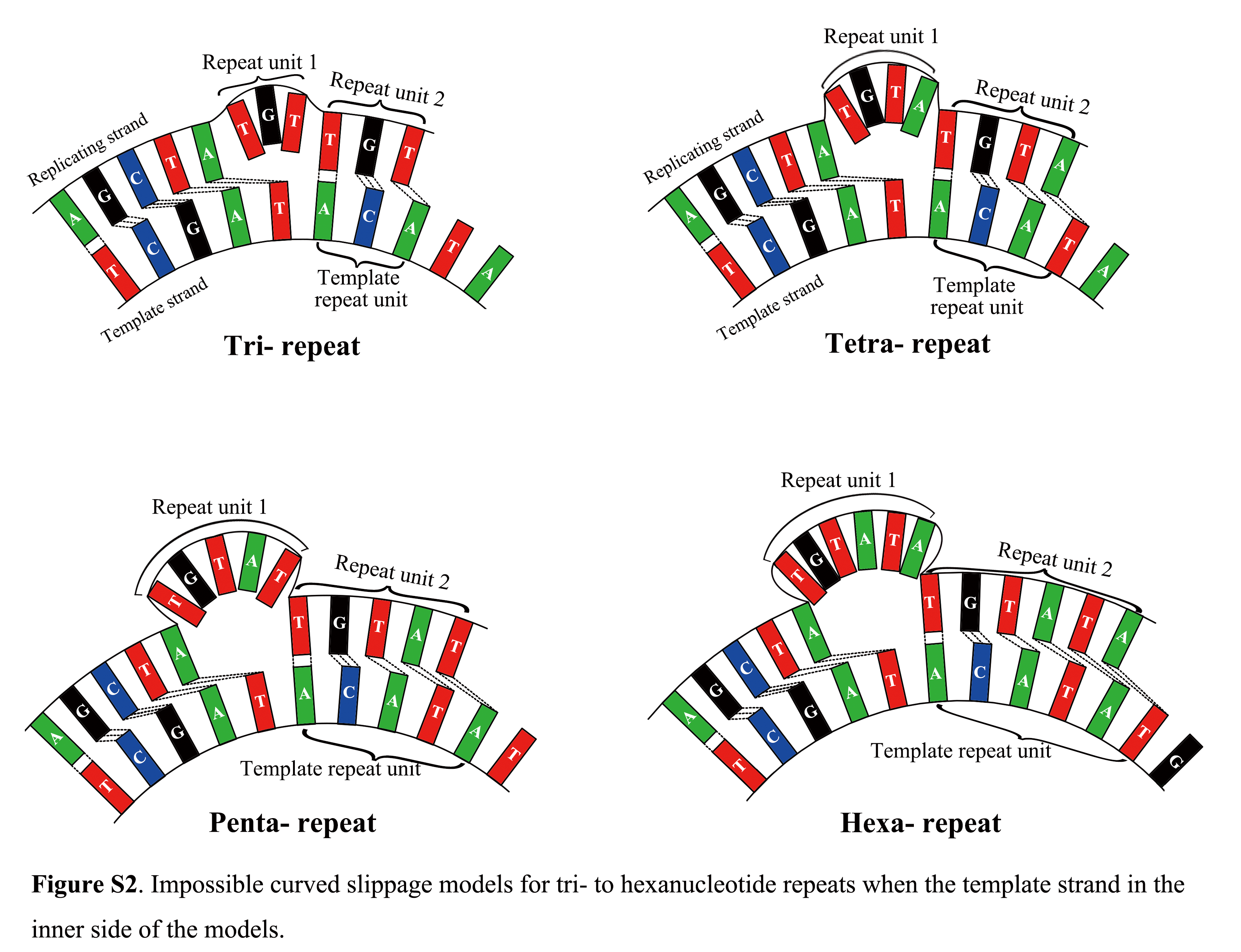

Supplement: Supplementary file 5 — Additional file 5. [file 12864_2020_6949_MOESM5_ESM.tiff]

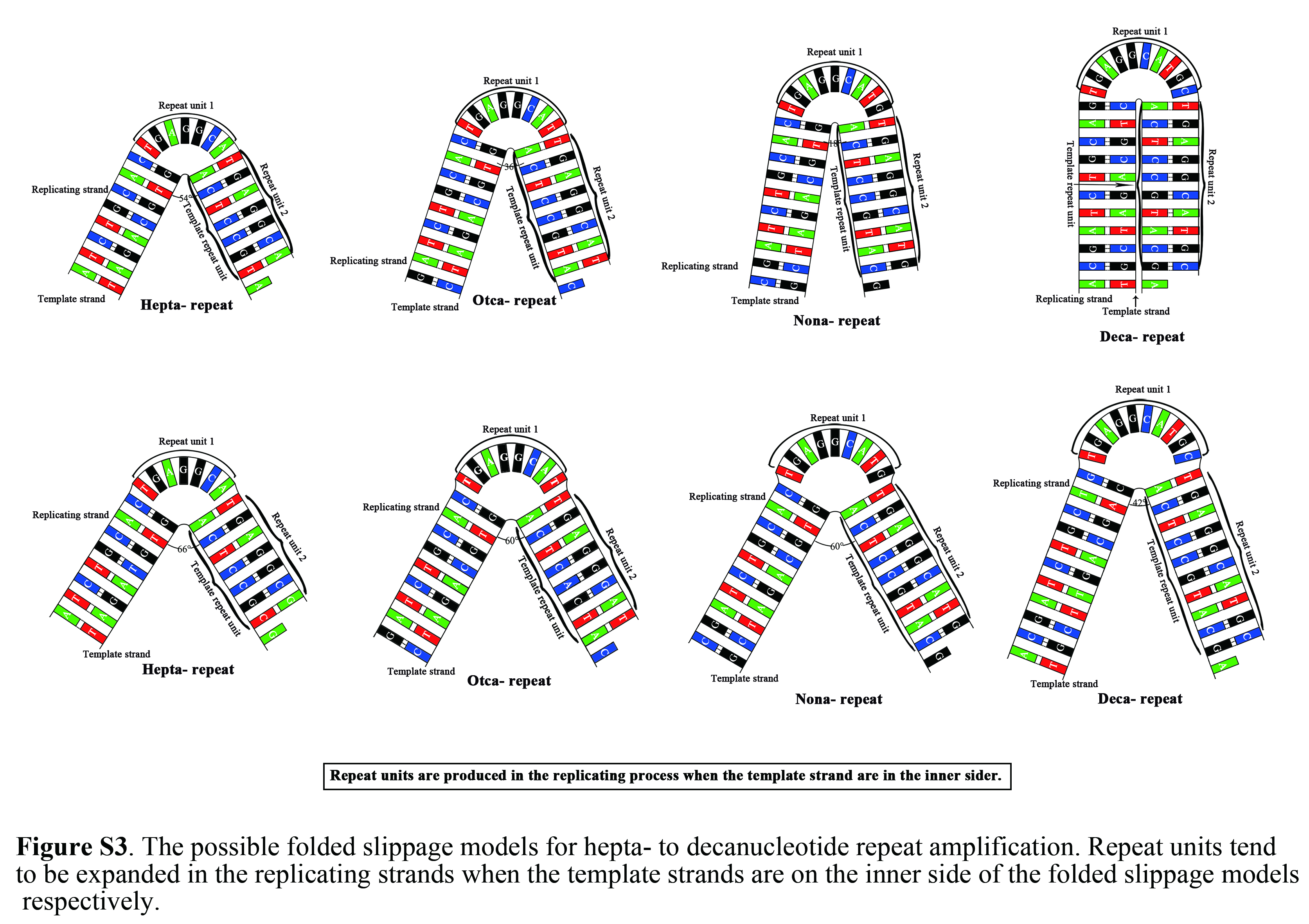

Supplement: Supplementary file 6 — Additional file 6. [file 12864_2020_6949_MOESM6_ESM.tiff]

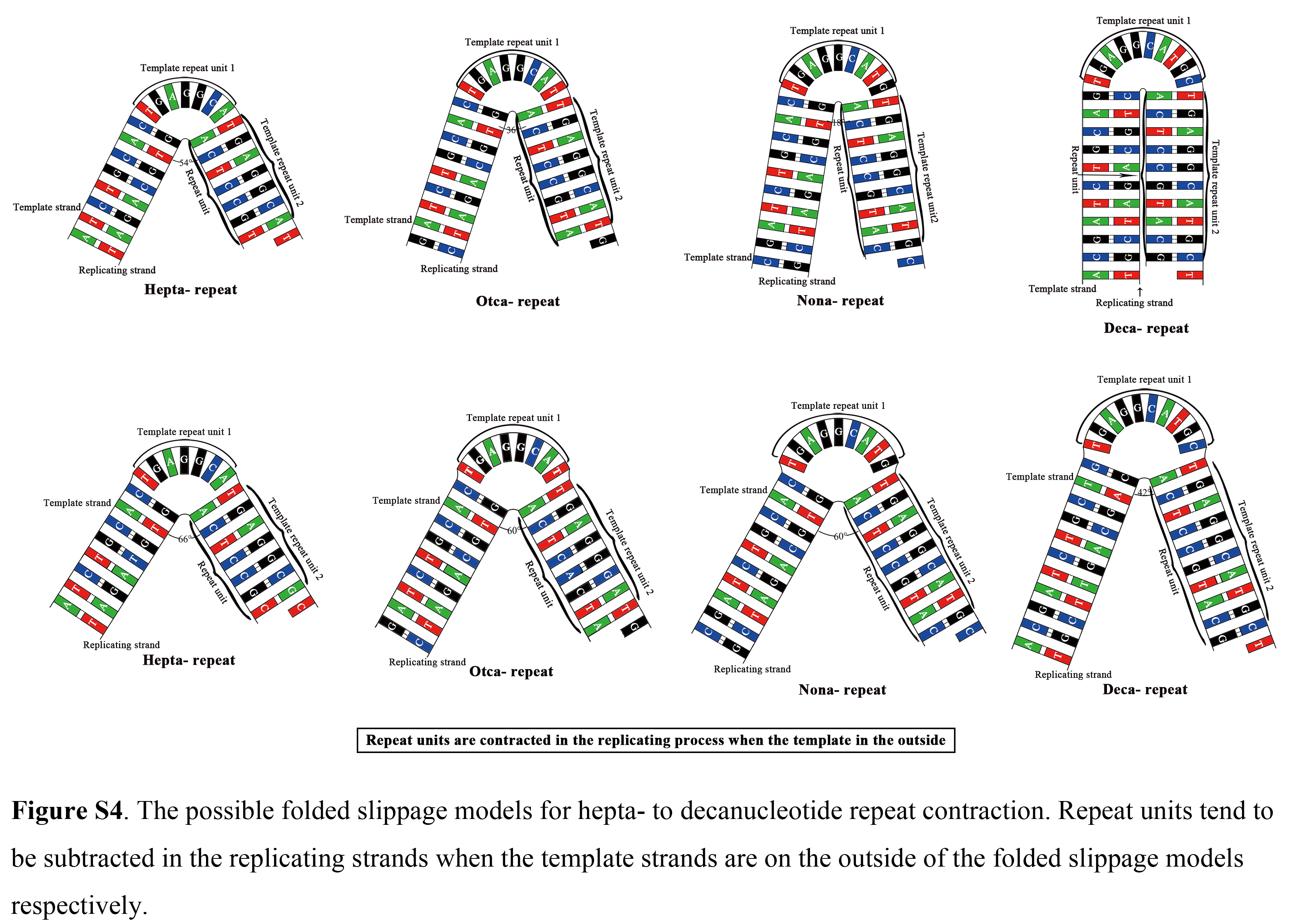

Supplement: Supplementary file 7 — Additional file 7. [file 12864_2020_6949_MOESM7_ESM.tiff]
